# Allogeneic hematopoietic stem cell transplantation for B‐cell lymphoma in Taiwan

**DOI:** 10.1002/cam4.6741

**Published:** 2023-11-28

**Authors:** Chun‐Hui Lee, Tzu‐Chien Lin, Ming Yao, Liang‐Tsai Hsiao, Bor‐Sheng Ko, Chia‐Jen Liu, Tsai‐Yun Chen

**Affiliations:** ^1^ Institute of Clinical Medicine, College of Medicine, National Cheng Kung University Tainan Taiwan; ^2^ Department of Oncology National Cheng Kung University Hospital, College of Medicine, National Cheng Kung University Tainan Taiwan; ^3^ Division of Hematology, Department of Internal Medicine National Cheng Kung University Hospital, College of Medicine, National Cheng Kung University Tainan Taiwan; ^4^ Division of Hematology, Department of Internal Medicine National Taiwan University Hospital, College of Medicine Taipei Taiwan; ^5^ Division of Hematology, Department of Internal Medicine Taipei Veterans General Hospital Taipei Taiwan; ^6^ Department of Hematological Oncology National Taiwan University Cancer Center Taipei Taiwan; ^7^ Institute of Emergency and Critical Care Medicine, School of Medicine National Yang‐Ming Chiao Tung University Taipei Taiwan; ^8^ Center for Cell Therapy National Cheng Kung University Hospital, College of Medicine, National Cheng Kung University Tainan Taiwan

**Keywords:** B‐cell lymphoma, complete remission, diffuse large B‐cell lymphoma, hematopoietic stem cell transplantation, relapse and refractory

## Abstract

Allogeneic hematopoietic stem cell transplantation (allo‐HSCT) is considered for patients with high‐risk B‐cell lymphoma and relapsed or refractory disease. This study aimed to analyze the long‐term follow‐up data of patients who underwent allo‐HSCT in Taiwan. This was a retrospective observational study using data from the Taiwan Society of Blood and Marrow Transplantation database. A total of 105 patients who underwent allo‐HSCT because of high‐risk, relapsed, or refractory disease between 2010 and 2019 were included. Forty‐five percent of the patients previously underwent autologous stem cell transplantation (ASCT). The median follow‐up duration was 18.6 months. The probability of 3‐year progression‐free survival and overall survival (OS) was 34.5% and 37%, respectively. The probability of 1‐year non‐relapse mortality was 31.4%, and the major cause was infection (75.8%). The multivariable analysis showed that not in remission at the time of transplantation and the absence of graft‐versus‐host disease (GVHD) were factors associated with inferior OS. The probability of 3‐year OS in patients with diffuse large B‐cell lymphoma who underwent allo‐HSCT and allo‐HSCT after ASCT was 40.2% and 25.2%, respectively. Allo‐HSCT could be a salvage therapeutic option for relapsed or refractory B‐cell lymphoma. Complete remission at the time of allo‐HSCT and the presence of GVHD are independent variables for overall survival.

## Graphical abstract

From our data, allo‐HSCT could be a salvage therapeutic option for relapsed or refractory B‐cell lymphoma. Complete remission at the time of allo‐HSCT and presence of GVHD are independent variables for overall survival.

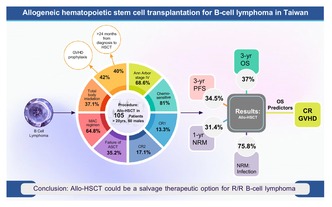

## 1 INTRODUCTION

The treatment of lymphoma has undergone revolutionary changes due to the discovery of novel drugs and advances in biology and diagnosis. Allogeneic hematopoietic stem cell transplantation (allo‐HSCT) still plays a vital role in treating certain non‐Hodgkin lymphoma subtypes.^1^, ^2^, ^3^, ^4^, ^5^, ^6^, ^7^, ^8^, ^9^ However, its efficacy may be limited by high non‐relapse mortality (NRM) due to the toxicity of the conditioning regimen, graft‐versus‐host disease (GVHD), and infection, particularly in patients who have been intensively pretreated. Despite these limitations, the literature has reported long‐term overall survival (OS) of 20%–50%.^10^, ^11^, ^12^

Therapeutics targeting immune checkpoints, molecular signaling pathways, tumor microenvironment, and epigenetic aberrations, in addition to cellular immunotherapy, comprise the new paradigm in the treatment of relapsed or refractory (R/R) diffuse large B‐cell lymphoma (DLBCL).^13^ Current risk stratification and treatment guidelines were established mostly based on research conducted in Western countries with a limited number of studies from Japan.^11^, ^12^, ^14^, ^15^ Tisagenlecleucel was approved for the third line of treatment of R/R DLBCL in Taiwan in 2019.^16^ Given the favorable outcomes of chimeric antigen receptor (CAR) T‐cell therapy, there is emerging uncertainty about the role of allo‐HSCT in treating R/R B‐cell lymphoma. Thus, this study aimed to analyze the long‐term follow‐up data of patients who underwent allo‐HSCT in Taiwan.

## 2 SUBJECTS AND METHODS

### 2.1 Data source

This was a retrospective observational study using data from the Taiwan Blood and Marrow Transplantation Registry (TBMTR). Since 2009, the Taiwan Society of Blood and Marrow Transplantation has been responsible for maintaining the TBMTR and recording the clinical information of blood and bone marrow transplant recipients. A total of 17 hospitals contributed to the Taiwan Society of Blood and Marrow Transplantation. The collection and analysis of data from the TBMTR were approved by the Institutional Review Board of each participating hospital. All experimental protocols were approved by the Institutional Review Board or Ethical Committee of the Taiwan Society of Blood and Marrow Transplantation. All procedures were conducted in accordance with applicable rules and regulations and the Declaration of Helsinki. Informed consent was obtained from all subjects or their legal guardians.

### 2.2 Patient selection

A total of 746 patients aged >20 years with B‐cell lymphoma who underwent HSCT between January 2010 and December 2019 were enrolled. Of the 746 patients, 639 were excluded due to undergoing autologous transplantation only, and two were lost to follow‐up. Finally, 105 patients were included for analysis. The patients underwent allo‐HSCT depending on the attending physicians' clinical judgment based on unfavorable prognostic factors, such as high‐risk B‐cell lymphoma, relapsed or refractory disease, and underlying medical status. The pathology report was classified according to the 2008 edition of the World Health Organization classification of lymphoma neoplasms. Data on prognosis‐relevant variables, including lymphoma histological types, age, International Prognostic Index, stage at diagnosis, disease status before allo‐HSCT, and conditioning regimens, were collected.

### 2.3 Allo‐HSCT regimens and definitions of response criteria

Conditioning regimens were classified as myeloablative conditioning (MAC) and non‐myeloablative conditioning (NMAC) regimens according to the definition of the International Workshop Criteria.^17^ MAC regimens include busulfan/cyclophosphamide and cyclophosphamide/total body irradiation, and NMAC regimens include a combination of fludarabine and melphalan/cyclophosphamide/busulfan. Status at transplantation was defined according to definitions of the International Working Group consensus response evaluation criteria in lymphoma.^18^ First complete remission (CR1) was defined as complete remission (CR) after primary chemotherapy. Primary refractory CR or relapsed and chemosensitive CR was defined as CR2 after salvage chemotherapy. Primary induction failure and sensitivity to chemotherapy were defined as partial response (PR) to treatment without reaching CR. Relapse or refractory before HSCT was defined as not in remission before HSCT. OS was defined as the time from transplant to death from any cause. Progression‐free survival (PFS) was defined as the time from allo‐HSCT treatment to relapse/progression or death from any cause. NRM includes all causes of death without disease progression/relapse occurring at any time after transplant. The primary endpoint of the study was OS, and the secondary endpoints were PFS and NRM.

### 2.4 Statistical methods

Discrete variables were analyzed using Fisher's exact test or χ^2^ test, whereas continuous variables were analyzed using the *t*‐test or Mann–Whitney *U*‐test. Probabilities of PFS and OS were estimated according to the Kaplan–Meier method, and the groups were compared using the log‐rank test. Associations between patient‐, disease‐, and transplantation‐related variables and outcomes of interest were evaluated using Cox proportional hazards regression. Factors, including B‐cell lymphoma subtypes, disease status at HSCT, age at HSCT, Ann Arbor stage at diagnosis, conditioning regimen, total body irradiation, treatment lines before HSCT, donor human leukocyte antigen match status, previous autologous stem cell transplant (ASCT), and time from diagnosis to HSCT, were considered in the analysis. Only variables with a P value less than 0.10 in univariate analysis were incorporated into the multivariable Cox proportional hazard regression analysis. In multivariate analysis, a P value of less than 0.05 indicates statistical significance. All statistical analyses and graphing were performed using GraphPad Prism statistical software, version 9 (GraphPad). A *p*‐value of <0.05 was considered statistically significant.

## 3 RESULTS

**FIGURE 1:**
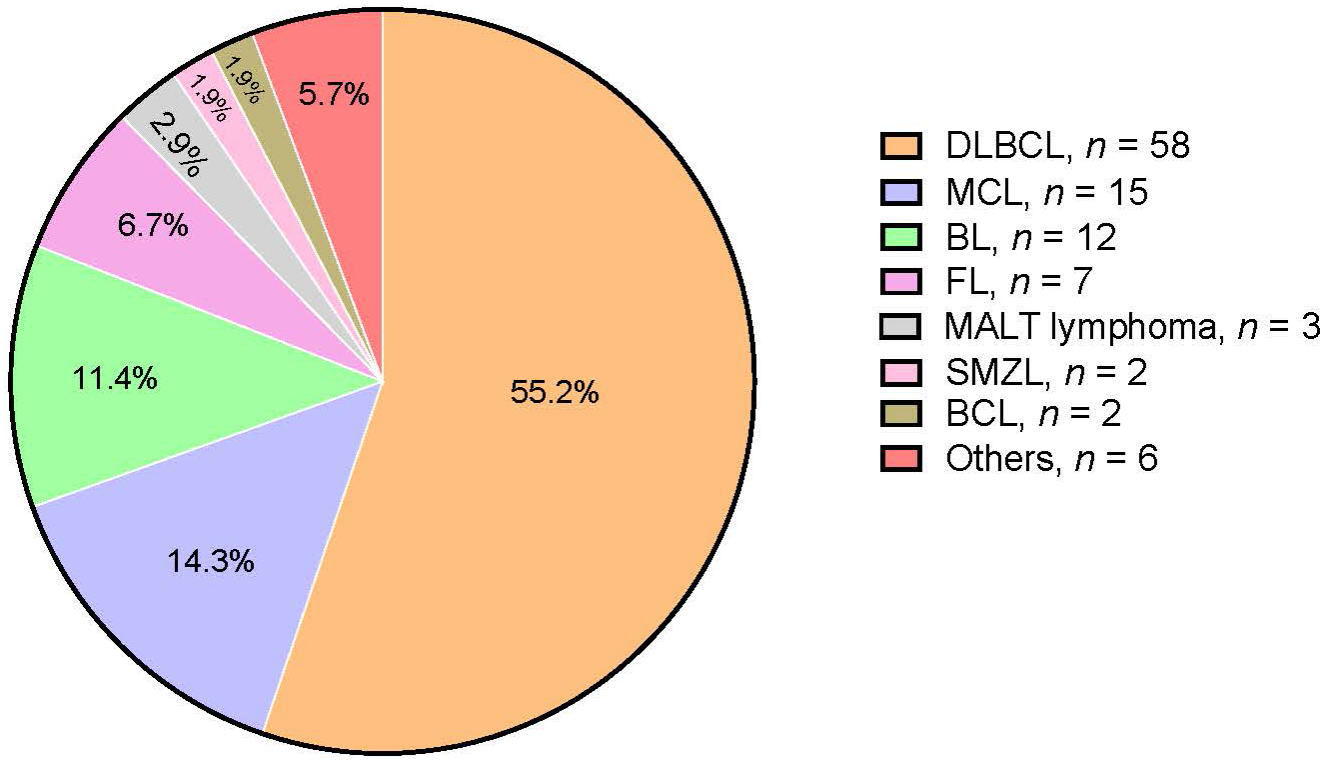
Subtypes of B‐cell lymphoma and numbers of patients with B‐cell lymphoma who received allogeneic stem cell transplant (allo‐HSCT) in Taiwan. DLBCL, diffuse large B‐cell lymphoma; BL, Burkitt lymphoma; MCL, mantle cell lymphoma; FL, follicular lymphoma; MALT lymphoma, mucosa‐associated lymphoid tissue lymphoma; SMZL, splenic marginal zone lymphoma; BCL, B‐cell lymphoma unclassifiable; Others: other subtypes of B‐cell lymphoma.

A total of 105 patients with B‐cell lymphoma who underwent allo‐HSCT were included in this study. Figure 1 shows the major histological subtypes of B‐cell lymphoma, which are DLBCL (*n* = 58), mantle cell lymphoma (*n* = 15), Burkitt lymphoma (*n* = 12), follicular lymphoma (*n* = 7), mucosa‐associated lymphoid tissue lymphoma (*n* = 3), splenic marginal zone lymphoma (*n* = 2), B‐cell lymphoma unclassifiable (*n* = 2), and others (other subtypes of B‐cell lymphoma) (*n* = 6). Of the 105 patients, 60 (57.1%) were males. The median age of the patients was 47 years. Most patients (68.6%) were in Ann Arbor stage IV at diagnosis. At the time of allo‐HSCT, 85 patients (81%) were chemosensitive. Before allo‐HSCT, 14 patients (13.3%) were in their CR1, and 18 (17.1%) were in their CR2 (Table 1). Transplantation‐related characteristics are shown in Table 2. Of the 105 patients, 37 (35.2%) underwent allo‐HSCT after failure of ASCT (ASCT‐allo‐HSCT), 68 (64.8%) received the MAC protocol as a conditioning regimen, 39 (37.1%) underwent total body irradiation, 42 (40%) received GVHD prophylaxis with rabbit anti‐thymocyte globulin, and 42 (40%) underwent allo‐HSCT at a time interval greater than 24 months from diagnosis to transplantation.

**TABLE 1:** Patients' baseline characteristics.

Characteristics	Number of patients (*n* = 105)
	*N* (%)
Sex	
Male	60 (57.1)
Age at transplantation, years	
Median (years)	47 (12–70)
≤20	6 (5.7)
20–30	11 (10.5)
30–40	18 (17.1)
40–50	32 (30.5)
50–60	31 (29.5)
60–70	7 (6.7)
B symptoms	
Absence	44 (41.9)
Presence	46 (43.8)
Unknown	15 (14.3)
Chemotherapy sensitivity	
Sensitive	85 (81.0)
Resistant	20 (19.0)
Ann Arbor stage at diagnosis	
I	4 (3.8)
II	10 (9.5)
III	17 (16.2)
IV	72 (68.6)
Unknown	2 (1.9)
Disease status at transplantation	
CR1	14 (13.3)
≥CR2	18 (17.1)
PR	24 (22.9)
Relapse, chemosensitive	24 (22.9)
Relapse/refractory	25 (23.8)

Abbreviations: CR, complete remission; IPI, International Prognostic Index; PR, partial remission.

**TABLE 2:** Transplantation‐related characteristics.

Characteristics	Number of patients (*n* = 105)
	*N* (%)
Conditioning regimen	
Myeloablative	68 (64.8)
Non‐myeloablative	37 (35.2)
With TBI	39 (37.1)
GVHD prophylaxis	
With ATG	42 (40.0)
Without ATG	63 (60.0)
aGVHD	46 (43.8)
With ATG	17 (37.0)
Without ATG	29 (63.0)
cGVHD	48 (45.7)
With ATG	18 (37.5)
Without ATG	30 (62.5)
Donor HLA match	
HLA‐identical sibling	55 (52.4)
Haploidentical	12 (11.4)
Matched unrelated	15 (14.3)
Mismatched unrelated	23 (21.9)
Mismatch 7/8	13 (12.4)
Mismatch 6/8	10 (9.5)
Graft type	
Bone marrow	3 (2.9)
Peripheral blood	102 (97.1)
Treatment line before HSCT	
1	26 (24.8)
2	40 (38.1)
≥3	38 (36.2)
Unknown	1 (0.9)
Previous ASCT	37 (35.2)
Dead	
Yes	62 (59.0)
No	43 (41.0)
Cause of death	
Relapse	29 (46.8)
Non‐relapse	33 (53.2)
Time from diagnosis to HSCT (month)	
≤6	9 (8.6)
6–12	33 (31.4)
12–18	14 (13.3)
18–24	7 (6.7)
>24	42 (40.0)

Abbreviations: aGVHD, acute graft‐versus‐host disease; ASCT, autologous stem cell transplantation; cGVHD, chronic graft‐versus‐host disease; GVHD, graft‐versus‐host disease; HSCT, hematopoietic stem cell transplantation; TBI, total body irradiation.

### 3.1 PFS and OS in all patients

**FIGURE 2:**
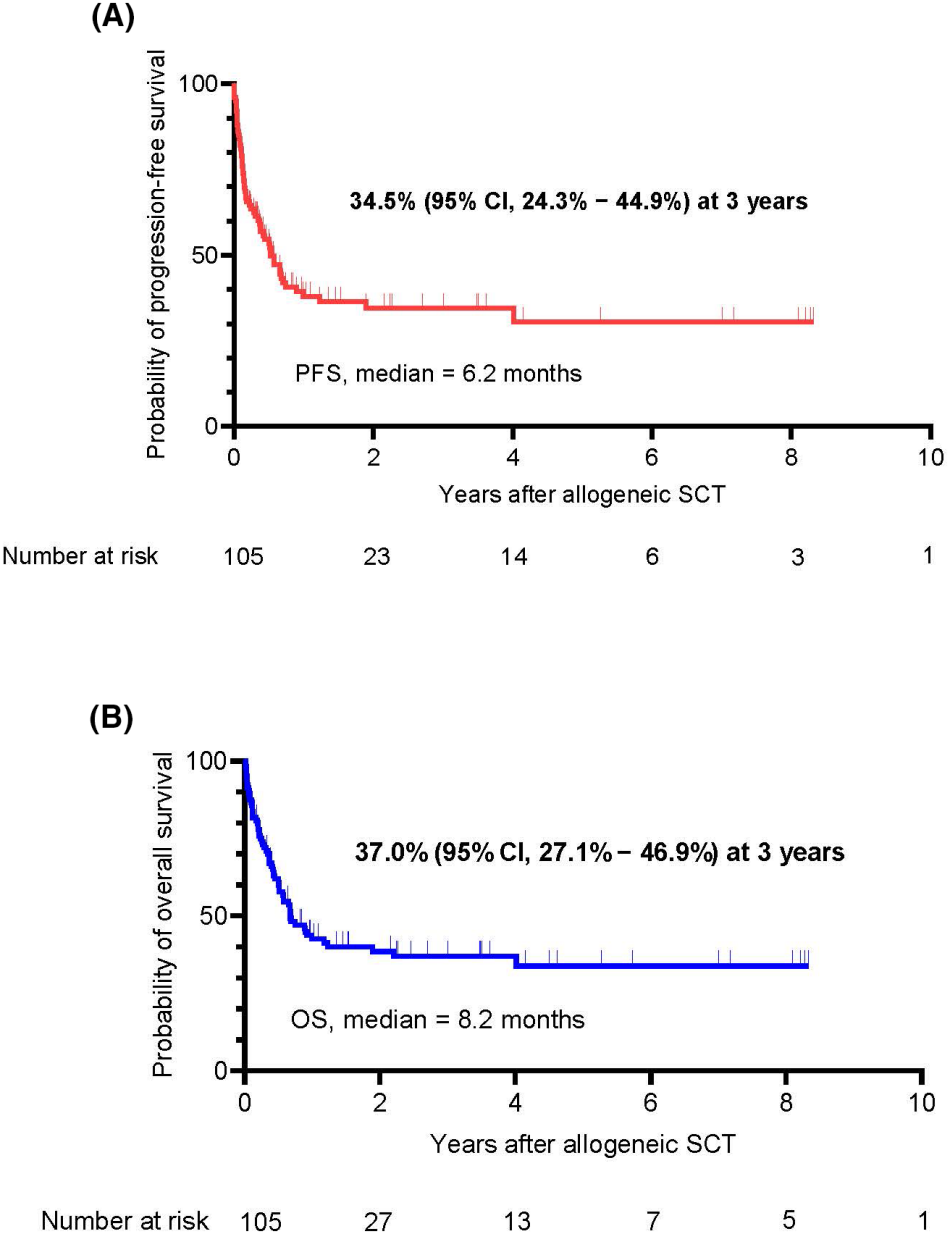
Kaplan–Meier plots of patients with B‐cell lymphoma who received allo‐HSCT. (A) Probability of progression‐free survival (PFS) in all patients. (B) Probability of overall survival (OS) in all patients.

**FIGURE 3:**
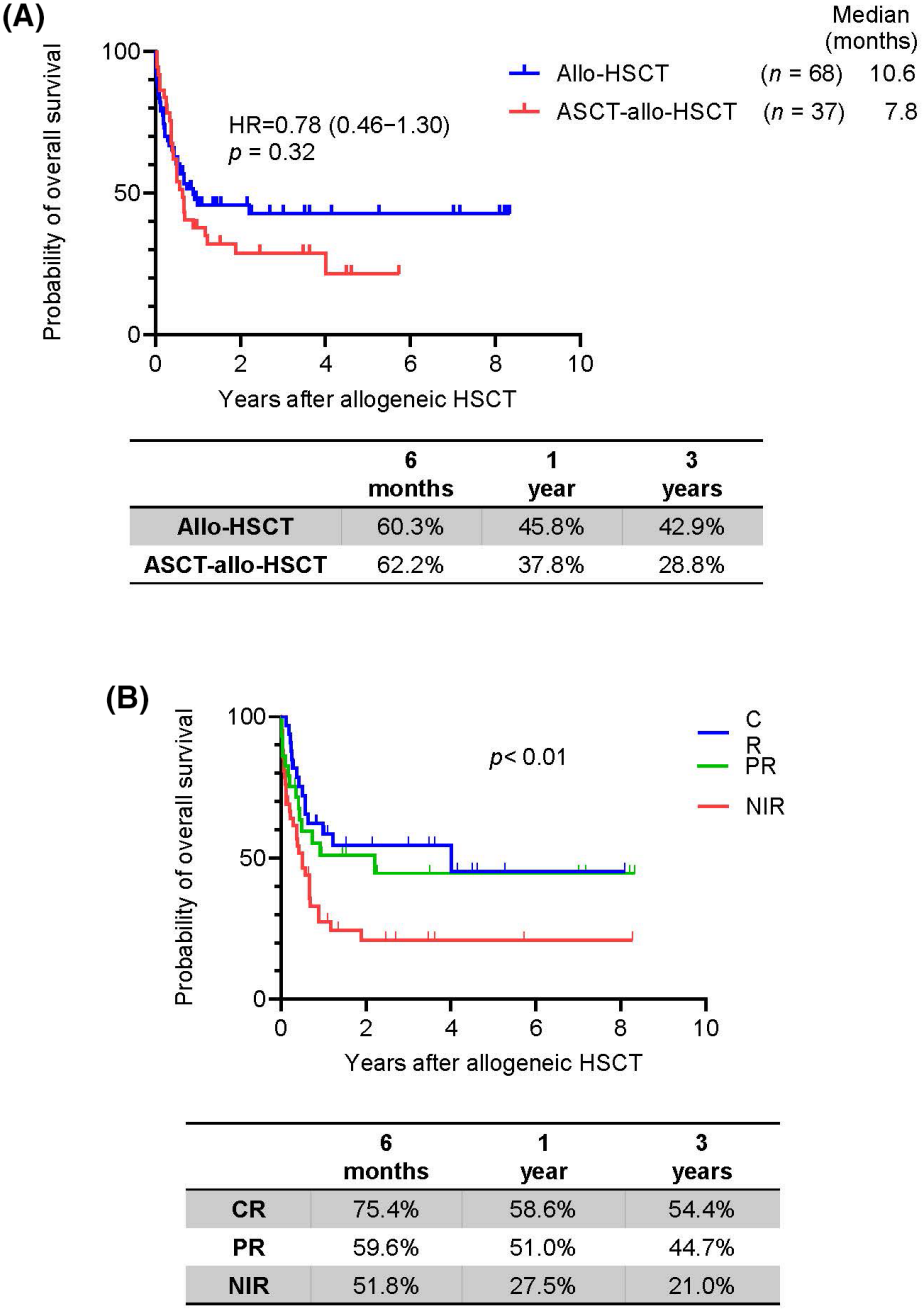
Kaplan–Meier plots of all patients who received allo‐HSCT. (A) OS and probability of 6‐month, 1‐year, and 3‐year survival in all patients after allo‐HSCT and allo‐HSCT after ASCT (ASCT‐allo‐HSCT). (B) OS according to disease status before allo‐HSCT.

The median follow‐up time of the survivors was 18.6 months (range, 0.2–120.8 months). The median PFS was 6.2 months, and the 3‐year PFS rate was 34.5% (95% confidence interval (CI): 24.3%–44.9%). The median OS was 8.2 months, and the 3‐year OS rate was 37.0% (95% CI: 27.1%–46.9%) (Figure 2A,B). The median OS was 10.6 months in patients who underwent allo‐HSCT and 7.8 months in patients who underwent ASCT‐allo‐HSCT (hazard ratio (HR) = 0.98, 95% CI: 0.46–1.30; *p* = 0.32) (Figure 3A). The probability of 1‐year and 3‐year OS was 45.8% and 42.9% in patients who underwent allo‐HSCT and 37.8% and 28.8% in patients who underwent ASCT‐allo‐HSCT (Figure 3A). The 3‐year OS rate in patients who achieved CR and PR was 54.4% (95% CI: 38.6%–70.2%) and 44.7% (95% CI: 25.9%–65.3%), respectively. The 3‐year OS rate of patients who were not in remission was 21.0% (95% CI: 6.5%–32.4%). Patients with a disease status of CR had significantly superior survival outcomes (*p* < 0.01, Figure 3B). The 1‐year NRM rate was 31.4%, and the major cause of NRM was infection (75.8%). In the multivariable analyses, not in remission at the time of allo‐HSCT (HR = 2.54, 95% CI: 1.45–4.48, *p* < 0.01) and the absence of GVHD (HR = 2.08, 95% CI: 1.19–3.61, *p* < 0.01) independently predicted low post‐allo‐HSCT survival (Tables S4 and S5).

### 3.2 Outcomes by lymphoma subtypes

**FIGURE 4:**
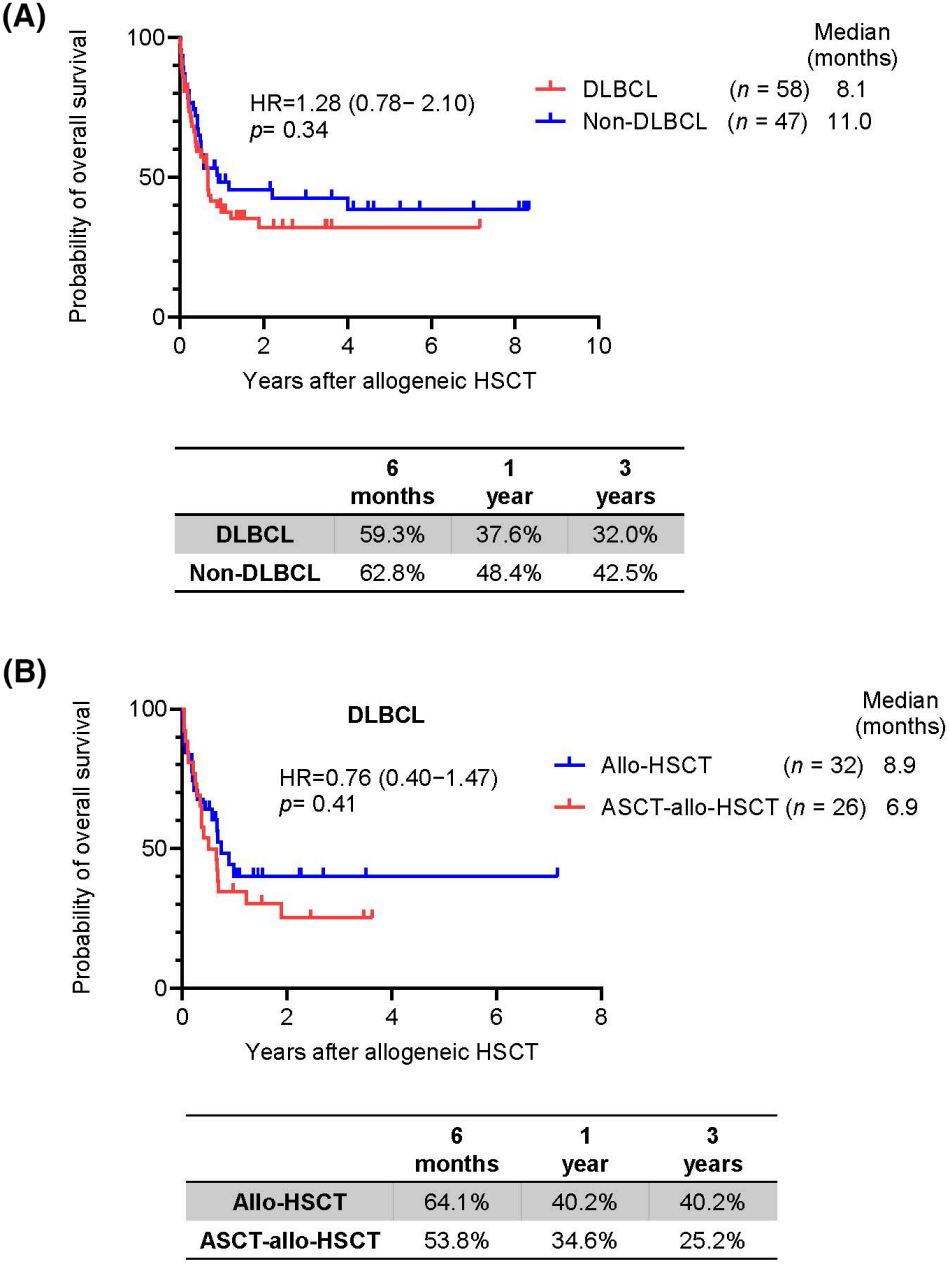
Kaplan–Meier plots stratified by subtypes of B‐cell lymphoma and sequence of stem cell transplantation. (A) OS and probability of 6‐month, 1‐year, and 3‐year survival between DLBCL and non‐DLBCL. (B) OS and probability of 6‐month, 1‐year, and 3‐year survival in patients with DLBCL who received allo‐HSCT (*n* = 32) and ASCT‐allo‐HSCT (*n* = 26).

The probability of OS was compared between patients with DLBCL and non‐DLBCL because of the low numbers of patients with other subtypes apart from DLBCL. The median OS was 8.1 months in patients with DLBCL and 11.0 months in patients with non‐DLBCL (HR = 1.28; *p* = 0.34). The 6‐month, 1‐year, and 3‐year OS rates were 59.3% (95% CI: 45.3%–70.8%), 37.6% (95% CI: 24.8%–50.4%), and 32.0% (95% CI: 19.4%–45.3%), respectively, in patients with DLBCL and 62.8% (95% CI: 47.1%–75.0%), 48.4% (95% CI: 33.0%–62.2%), and 42.5% (95% CI: 27.3%–56.9%), respectively, in patients with non‐DLBCL (Figure 4A). Among patients with DLBCL, 32 patients (55%) underwent allo‐HSCT, and 26 patients (45%) underwent ASCT‐allo‐HSCT (Tables S1 and S2). The median OS was 8.9 months in patients who underwent allo‐HSCT and 6.9 months in patients who underwent ASCT‐allo‐HSCT (HR = 0.76; *p* = 0.41). The probability of 3‐year OS was 40.2% in patients who underwent allo‐HSCT and 25.2% in patients who underwent ASCT‐allo‐HSCT (Figure 4B). There was no significant survival predictor among this subset of group (Tables S6 and S7).

### 3.3 Subset analysis

An exploratory subset analysis was performed to assess the outcomes of patients with DLBCL who underwent allo‐HSCT and ASCT‐allo‐HSCT. Demographic and transplantation‐related characteristics are shown in Tables S1 and S2. Among patients who underwent allo‐HSCT, more patients had a disease status of CR1 at the time of transplantation. At 1 year, the cumulative incidence rates of relapse mortality and NRM were 34.3% and 18.8%, respectively (Table S3). Among patients who underwent ASCT‐allo‐HSCT, the 1‐year relapse mortality and NRM rates were 19.1% and 46.2%. The major cause of NRM was infection (75%) (Table S3). There was no survival prognostic significance of time interval (>1 year vs. ≤1 year) in terms of ASCT to relapse and ASCT to allo‐HSCT in DLBCL patients who received ASCT‐allo‐HSCT (Figure S1). In univariable and multivariable analyses, no significant predictor of OS was observed (Tables S6 and S7).

## 4 DISCUSSION

High‐dose chemotherapy with ASCT is an effective salvage treatment for patients with R/R B‐cell lymphoma. Nevertheless, the disease may recur in some patients, and their outcomes are fortuneless.^19^, ^20^, ^21^, ^22^ In this study, the data of 105 Taiwanese patients who underwent allo‐HSCT between 2010 and 2019 were analyzed. The study findings confirmed that allo‐HSCT is an effective salvage treatment option for B‐cell lymphoma, with 3‐year PFS and OS rates of 34.5% and 37% and a 1‐year NRM rate of 31.4%. These findings are similar to those reported in previous studies.^10^, ^18^, ^21^ A retrospective cohort study including 68 patients who underwent allo‐HSCT in Kyoto Stem Cell Transplantation Group hospitals showed that the 4‐year OS and PFS rates in patients with DLBCL who underwent allo‐HSCT were 23% and 20%, respectively.^14^ Herrera et al^23^ reported that the 3‐year incidence of OS and relapse in 118 patients with chronic lymphocytic leukemia or small lymphocytic lymphoma with Richter's transformation who underwent allo‐HSCT were 52% and 30%. Another retrospective study on 141 patients with follicular lymphoma who underwent allo‐HSCT due to relapse after ASCT showed 5‐year OS and PFS rates of 49.1% and 47.4%.^15^ In an analysis of 51 patients with relapsed and refractory mantle cell lymphoma who underwent allo‐HSCT, the median OS was 65 months.^12^ Around 30%–40% of DLBCL ASCT recipients may experience relapse or recurrence.^6^, ^8^ In our subgroup DLBCL analysis, the result showed a low probability of survival in patients who underwent ASCT‐allo‐HSCT, 3‐year OS rate 42.9% (allo‐HSCT) versus 28.8% (ASCT‐allo‐HSCT). Of note, 18 patients (69.2%) had disease recurrence 24 months after ASCT. Despite the poor prognosis of patients with DLBCL after ASCT, some patients are still appropriate for allo‐HSCT. In high‐risk patients, allo‐HSCT could be a treatment option based on the clinical judgment of the attending physician. In this study, the 1‐year relapse mortality rate was 34.3% in patients with DLBCL who underwent allo‐HSCT and 19.1% in those who underwent ASCT‐allo‐HSCT. This finding shows that patients who underwent treatment with allo‐HSCT directly may have worse prognostic factors or aggressive disease status at diagnosis.

The impact of achieving CR before allo‐HSCT remains the cornerstone of survival. This study showed that the 3‐year OS rate in patients who achieved CR was 54.4%, whereas the 3‐year OS rate in patients whose diseases were not in remission was 21.0%. These results are consistent with those reported by Bento et al. who reported that active disease at allo‐HSCT (HR = 1.95; *p* = 0.039) was the independent variable for PFS and OS.^10^ Moreover, two cohort studies in Japan revealed that advanced disease status at allo‐HSCT was associated with a higher rate of relapse and progression and a lower rate of PFS.^14^, ^15^

The outcomes of allo‐HSCT are heavily influenced by NRM. This study showed a 1‐year NRM rate of 31.4% in all patients. Allo‐HSCT using MAC regimens significantly reduces relapse risk but at the expense of higher NRM due to GVHD or infection.^9^, ^24^, ^25^, ^26^, ^27^, ^28^, ^29^ In this study, the major cause of NRM was infection (75.8%). This result may be due to the fact that 68 patients (64.8%) received MAC regimens, and patients with acute GVHD accounted for 43.8% of our patients.

In recent years, significant efforts have been made to develop a broad spectrum of novel targeted therapies in B‐cell neoplasms. Monoclonal antibodies, antibody–drug conjugates (ADC), bispecific T‐cell‐engaging antibodies, CAR T cells, immune checkpoint inhibitors, and novel small molecules have been developed as immune‐based therapies. Polatuzumab vedotin and tafasitamab are recommended by NCCN guidelines as the second line of treatment in R/R DLBCL who have no intention to proceed to transplant.^30^, ^31^ Anti‐CD20 × CD3 antibodies, epcoritamab, glofitamab are also approved in patients with disease progression after transplant or CAR T‐cell therapy.^32^, ^33^ Loncastuximab tesirine, an anti‐CD19 conjugated ADC, received accelerated FDA approval for treating R/R DLBCL after two or more lines of therapy, based on phase II multicenter LOTIS‐2 trial results.^34^, ^35^ The most noteworthy is CAR T‐cell therapy. Overall, the data indicate that CD19‐targeted CAR T cells can induce prolonged remissions in patients with B‐cell malignancies, frequently with minimal long‐term toxicity, and are likely curative in a subset of patients.^16^, ^36^, ^37^, ^38^, ^39^, ^40^, ^41^, ^42^ Despite the high rate of CRs seen with CAR T‐cell therapies, only 30%–40% of patients achieve durable remissions.^42^, ^43^ Allo‐HSCT remains the curable potential in patients with R/R B‐cell lymphoma.^17^, ^18^, ^19^, ^20^, ^21^ Besides, relapse following CAR T‐cell therapy had a poor prognosis and was regarded as the emergence of resistance. Significant patterns of CAR T‐cell therapy resistance have been investigated. The lack of response to CAR T‐cells (primary resistance, antigen‐positive relapse) results from impaired death receptor signaling and dysfunctional CAR T cells. Loss of CD19 antigen and low‐quality CAR T‐cell expansion or T‐cell exhaustion contribute to disease progression (secondary resistance, antigen‐negative relapse) following the response.^44^, ^45^ After CAR T‐cell therapy, there are limited treatment options, and persistent cytopenia can impede salvage therapy or the opportunity to participate in clinical trials.^46^, ^47^ In addition, little evidence suggests that allo‐HSCT may be capable of rescuing a subset of CAR T‐cell therapy recipients with persistent or recurrent disease.^48^ Thus far, both allo‐HSCT and CAR T‐cell therapy can provide durable remission in a subset of patients with R/R B‐cell lymphoma. Clinicians are attempting to determine whether the two treatments are complementary to one another or therapeutic alternatives. In the literature, few studies have directly compared the outcomes of allo‐HSCT and CAR T‐cell therapy treatment in B‐cell lymphoma. Both treatments induce immunological responses against lymphoma cells but differ in many ways. These variations support the individual role of each therapy within treatment algorithms.^49^ Moreover, one major drawback of CAR T‐cell therapy is the availability due to disparities in healthcare systems, regulatory approval processes, and cost. Consequently, risk factor‐specific prospective studies are mandated to characterize better the optimal sequence of allo‐HSCT, cellular therapy, and other authorized novel therapies. This study has some limitations. First, this was a registry‐based study and lack of information in terms of germinal center B cell, non‐germinal center B cell, and double/triple expressor of DLBCL. Second, B‐cell lymphoma is heterogeneous due to the rarity of the disease and eligibility for transplantation, which is another limitation. Third, the initial time of enrollment was approximately 12 years ago, thus introducing various inherent confounding factors into our analysis. Notwithstanding these constraints, this study provides analyses based on patient characteristics and treatment patterns that investigators can use as benchmarks for future prospective studies.

In conclusion, this retrospective multicenter study demonstrated that allo‐HSCT could be a salvage therapeutic option for R/R B‐cell lymphoma. CR at the time of allo‐HSCT and the presence of GVHD could be OS prognostic factors.

## AUTHOR CONTRIBUTIONS

**Chun‐Hui Lee:** Conceptualization (lead); data curation (lead); formal analysis (lead); methodology (lead); resources (lead); software (lead); validation (lead); visualization (lead); writing – original draft (lead); writing – review and editing (lead). **Tzu‐Chien Lin:** Data curation (lead); formal analysis (lead); methodology (lead); resources (lead); software (lead); writing – original draft (lead). **Ming Yao:** Investigation (equal); project administration (equal); resources (equal). **Liang‐Tsai Hsiao:** Investigation (equal); project administration (equal); resources (equal). **Bor‐Sheng Ko:** Investigation (equal); project administration (equal); resources (equal). **Chia‐Jen Liu:** Investigation (equal); project administration (equal); resources (equal). **Tsai‐Yun Chen:** Conceptualization (lead); data curation (lead); formal analysis (lead); methodology (lead); resources (lead); supervision (lead); validation (lead); visualization (lead); writing – original draft (lead); writing – review and editing (lead).

## FUNDING INFORMATION

This research received no specific grant from any funding agency in the public, commercial, or not‐for‐profit sectors.

## CONFLICT OF INTEREST STATEMENT

The authors declare no competing interests.

## Supporting information


Figure S1.

Table S1.

Table S2.

Table S3.

Table S4.

Table S5.

Table S6.

Table S7.

## Data Availability

The final analysis dataset is available upon request to the Working Party chair.
