# Supplementary figures and images for: Allogeneic hematopoietic stem cell transplantation for B‐cell lymphoma in Taiwan
Source: Cancer Med. 2023 Nov 28;12(24):21761–9. doi: 10.1002/cam4.6741 (PMC10757116; doi:10.1002/cam4.6741)

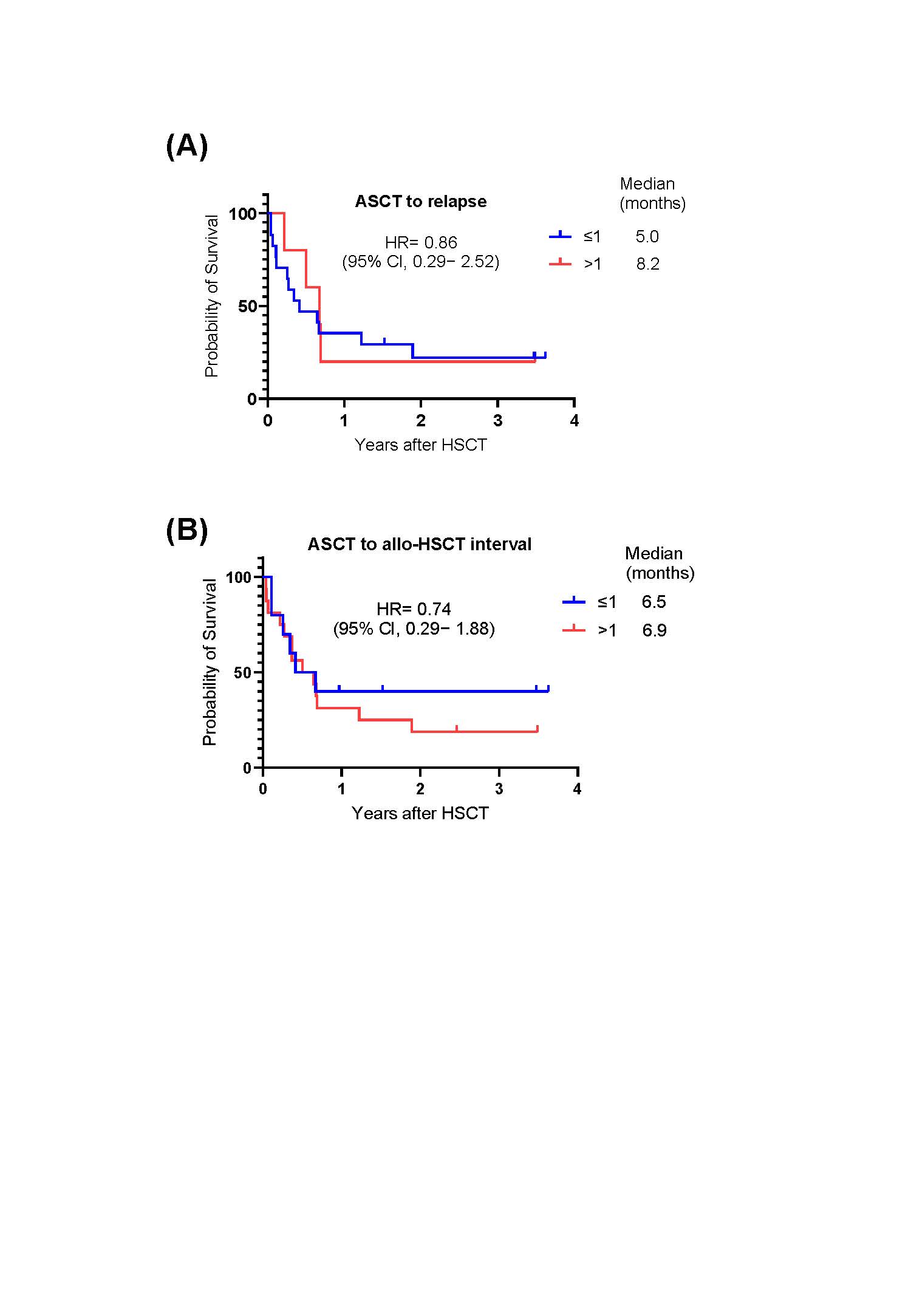

Supplement: Supplementary file 1 — Figure S1. [file CAM4-12-21761-s005.jpg]
