# Supplementary material for: Allogeneic hematopoietic stem cell transplantation for B‐cell lymphoma in Taiwan
Source: Cancer Med. 2023 Nov 28;12(24):21761–9. doi: 10.1002/cam4.6741 (PMC10757116; doi:10.1002/cam4.6741)
Supplement: Supplementary file 2 — Table S1. [file CAM4-12-21761-s004.pdf]

Supplementary Table S1. Baseline characteristics of patients with DLBCL (n = 58).

Characteristics	Number of patients, *n* (%)		
	**ASCT-allo-HSCT (*n* = 26)**	**Allo-HSCT (*n* = 32)**	***p***
**Sex**			0.24
Male	17 (65.4)	16 (50)	
**Age at transplantation, years**			0.94
**Median (years)**	46 (18–59)	45 (11–61)	
≤20	2 (7.7)	3 (9.3)	
20–30	3 (11.5)	4 (12.5)	
30–40	4 (15.4)	6 (18.8)	
40–50	7 (26.9)	8 (25)	
50–60	10 (38.5)	10 (31.3)	
>60	0 (0)	1 (3.1)	
**B symptoms**			0.44
Absence	9 (34.6)	12 (37.5)	
Presence	16 (61.5)	16 (50)	
Unknown	1 (3.9)	4 (12.5)	
**Chemotherapy sensitivity**			0.75
Sensitive	22 (84.6)	28 (87.5)	
Resistant	4 (15.4)	4 (12.5)	
**Ann Arbor stage at diagnosis**			0.52
I	2 (7.7)	1 (3.1)	
II	2 (7.7)	6 (18.7)	
III	7 (26.9)	6 (18.7)	
IV	15 (57.7)	19 (59.5)	
**IPI score**			0.16
0-1	2 (7.7)	0 (0)	
2	2 (7.7)	1 (3.1)	
3	3 (11.5)	1 (3.1)	
4	0 (0)	2 (6.3)	
Unknown	19 (73.1)	28 (87.5)	
**Disease status at transplantation**			<0.01
CR1	2 (7.7)	5 (15.6)	

≥CR2	8 (30.8)	0 (0)
PR	4 (15.4)	12 (37.5)
Relapse, chemosensitive	7 (26.9)	6 (18.8)
Relapse/refractory	5 (19.2)	9 (28.1)

ASCT autologous stem cell transplantation, *ASCT-allo-HSCT* allo-HSCT with previous ASCT, *IPI* International Prognostic Index*, CR* complete remission, *PR* partial remission
