# Supplementary material for: Allogeneic hematopoietic stem cell transplantation for B‐cell lymphoma in Taiwan
Source: Cancer Med. 2023 Nov 28;12(24):21761–9. doi: 10.1002/cam4.6741 (PMC10757116; doi:10.1002/cam4.6741)
Supplement: Supplementary file 3 — Table S2. [file CAM4-12-21761-s002.pdf]

Supplementary Table S2. Transplantation-related characteristics of patients with DLBCL (n = 58).

**Characteristics**	**Number of patients, *n* (%)**		
	**ASCT-allo-HSCT (*n* = 26)**	**Allo-HSCT (*n* = 32)**	***p***
**Conditioning regimen**			0.94
Myeloablative	16 (61.5)	20 (62.5)	
Non-myeloablative	10 (38.5)	12 (37.5)	
**With TBI**	7 (26.9)	13 (40.6)	0.27
**GVHD prophylaxis**			<0.05
With ATG	4 (15.4)	13 (40.6)	
Without ATG	22 (84.6)	19 (59.4)	
**aGVHD**	12 (46.2)	16 (50.0)	0.77
With ATG	3 (75)	6 (46.2)	
Without ATG	9 (40.9)	10 (52.6)	
**cGVHD**	8 (30.8)	16 (50.0)	0.14
With ATG	2 (50)	6 (46.2)	
Without ATG	6 (27.3)	11 (57.9)	
**Donor HLA match**			
HLA-identical sibling	16 (61.5)	15 (46.9)	<0.05
Haploidentical	6 (23.1)	2 (6.3)	
Matched unrelated	1 (3.8)	4 (12.5)	
Mismatched unrelated	3 (11.5)	11 (34.3)	
Mismatch 7/8	2 (7.7)	6 (54.5)	
Mismatch 6/8	1 (3.8)	5 (45.5)	
**Graft type**			0.36
Bone marrow	0 (0)	1 (3.1)	
Peripheral blood	26 (100)	31 (96.9)	
**Treatment line before HSCT**			<0.01
1	0 (0)	15 (46.9)	
2	14 (53.8)	7 (21.9)	
≥3	12 (46.2)	9 (28.1)	
Unknown	0 (0)	1 (3.1)	
**Dead**			0.12
Yes	19 (73.1)	17 (53.1)	

No	7 (26.9)	15 (46.9)	
**Cause of death**			<0.05
Relapse	6 (31.6)	11 (64.7)	
Non-relapse	13 (68.4)	6 (35.3)	
**Time from diagnosis to allo-HSCT (month)**			<0.01
≤6	0 (0)	4 (12.5)	
6–12	3 (11.5)	14 (43.8)	
12–18	2 (7.7)	6 (18.8)	
18–24	3 (11.5)	1 (3.1)	
>24	18 (69.2)	7 (21.9)	

ASCT autologous stem cell transplantation, *ASCT-allo-HSCT* allo-HSCT with previous ASCT, *TBI* total body irradiation, *GVHD* graft-versus-host disease, *aGVHD* acute graft-versus-host disease, *cGVHD* chronic graft-versus-host disease, *HSCT* hematopoietic stem cell transplantation
