# Supplementary material for: Allogeneic hematopoietic stem cell transplantation for B‐cell lymphoma in Taiwan
Source: Cancer Med. 2023 Nov 28;12(24):21761–9. doi: 10.1002/cam4.6741 (PMC10757116; doi:10.1002/cam4.6741)
Supplement: Supplementary file 4 — Table S3. [file CAM4-12-21761-s006.pdf]

Supplementary Table S3. Survival outcomes in patients with DLBCL ($n = 58$).

**Characteristics**	**Number of patients, *n* (%)**	
	**ASCT-allo-HSCT (*n* = 26)**	**Allo-HSCT (*n* = 32)**
**1-year mortality**	65.3%	53.1%
**1-year NRM**	46.2%	18.8%
Major cause	Infection (75%)	Infection (66.7%)

ASCT autologous stem cell transplantation, *ASCT-allo-HSCT* allo-HSCT with previous ASCT, *NRM* non-relapse mortality, *DLBCL* diffuse large B-cell lymphoma
