# Supplementary material for: Allogeneic hematopoietic stem cell transplantation for B‐cell lymphoma in Taiwan
Source: Cancer Med. 2023 Nov 28;12(24):21761–9. doi: 10.1002/cam4.6741 (PMC10757116; doi:10.1002/cam4.6741)
Supplement: Supplementary file 5 — Table S4. [file CAM4-12-21761-s001.pdf]

Supplementary Table S4. Univariable analysis for prognostic factors of survival to the end of follow-up.

Variables	Median survival (months)	*p*
**Lymphoma subtypes**		
DLBCL	8.1	0.34
Non-DLBCL	11.0	
**Disease status at allo-HSCT**		
CR	48.2	<0.01
PR	26.4	
NIR	6.2	
**Age at allo-HSCT, years**		
≤40	5.1	0.27
>40	8.1	
**Ann Arbor stage at diagnosis**		
I-II	6.9	0.05
III-IV	8.3	
**Conditioning regimen**		
Myeloablative	6.0	0.36
Non-myeloablative	10.7	
**TBI**		
TBI	18.4	0.47
No TBI	10.7	
**Treatment line before allo-HSCT**		
1	NR	0.08
2	6.9	
≥ 3	6.2	

**Donor HLA match**		
HLA-identical sibling	6.9	0.15
Haploidentical	10.6	
Matched unrelated	26.4	
Mismatched unrelated	NR	
**GVHD**		
Any GVHD	11.9	<0.01
No GVHD	4.4	
**ATG**		
ATG use	13.0	0.66
No ATG use	8.0	

DLBCL diffuse large B-cell lymphoma, *TBI* total body irradiation, *HSCT* hematopoietic stem cell transplantation, *HLA* human leukocyte antigen, *CR* complete remission, *PR* partial remission, *NIR* not in remission, *NR* not reached, *GVHD* graft-versus-host disease, *ATG* anti-thymocyte globulin
