# Supplementary material for: Allogeneic hematopoietic stem cell transplantation for B‐cell lymphoma in Taiwan
Source: Cancer Med. 2023 Nov 28;12(24):21761–9. doi: 10.1002/cam4.6741 (PMC10757116; doi:10.1002/cam4.6741)
Supplement: Supplementary file 6 — Table S5. [file CAM4-12-21761-s008.pdf]

Supplementary Table S5. Prognostic factors for the overall survival in the multivariable analysis.

Variables	*N*	Hazard ratio	95% CI Lower limit	95% CI Upper limit	*p*
**Disease status at allo-HSCT**					
CR and PR	56	1			
Not in remission	49	2.54	1.45	4.48	<0.01
**Ann Arbor stage at diagnosis**					
III-IV	91	1			
I-II	14	1.90	0.88	3.73	0.08
**Treatment line before HSCT**					
1	26	1			
>1	78	1.03	0.46	2.24	0.95
**GVHD**					
Yes (any grade)	70	1			
No	35	2.08	1.19	3.61	<0.01

HSCT hematopoietic stem cell transplantation, *GVHD* graft-versus-host disease, *DLBCL* diffuse large B-cell lymphoma, *CR* complete remission, *PR* partial remission
