# Supplementary material for: Allogeneic hematopoietic stem cell transplantation for B‐cell lymphoma in Taiwan
Source: Cancer Med. 2023 Nov 28;12(24):21761–9. doi: 10.1002/cam4.6741 (PMC10757116; doi:10.1002/cam4.6741)
Supplement: Supplementary file 7 — Table S6. [file CAM4-12-21761-s003.pdf]

Supplementary Table S6. Prognostic factors for the overall survival in the univariable analysis (DLBCL).

Variables	3-year OS (%)	*p*
**Previous ASCT**		0.41
No	40.2	
Yes	25.2	
**Treatment line**		0.20
≤2	37.6	
>2	22.3	
**Diagnosis to allo-HSCT**		0.10
≤1 year	50.1	
>1 year	21.7	
**Disease status at allo-HSCT**		0.05
Non-relapse/refractory	27.6	
Relapse/refractory	18.4	
**Diagnosis to ASCT**		0.68
≤1 year	33.3	
>1 year	21.4	
**ASCT to relapse**		0.68
≤1 year	32.1	
>1 year	20.0	
**ASCT to allo-HSCT interval**		0.53
≤1 year	40.0	
>1 year	18.8	

OS overall survival, *DLBCL* diffuse large B-cell lymphoma, *ASCT* autologous stem cell transplantation, *HSCT* hematopoietic stem cell transplantation
