# Supplementary material for: Allogeneic hematopoietic stem cell transplantation for B‐cell lymphoma in Taiwan
Source: Cancer Med. 2023 Nov 28;12(24):21761–9. doi: 10.1002/cam4.6741 (PMC10757116; doi:10.1002/cam4.6741)
Supplement: Supplementary file 8 — Table S7. [file CAM4-12-21761-s007.pdf]

Supplementary Table S7. Prognostic factors for the overall survival in the multivariable analysis (DLBCL).

Variables	Hazard ratio (95% CI)	*p*-value
**Diagnosis to HSCT**		
≤1 year	Reference	
>1 year	1.45 (0.61–3.59)	0.41
**Disease status at allo-HSCT**		
Non-relapse/refractory	Reference	
Relapse/refractory	1.74 (0.87–3.46)	0.12

DLBCL: diffuse large B-cell lymphoma; ASCT: autologous stem cell transplantation; HSCT: hematopoietic stem cell transplantation.
